# Expanding the Spectrum of Src-Family Kinase-Related Autoinflammatory Diseases: Monogenic Vasculitis Caused By Germline Pathogenic Variants in *HCK* and *FGR*

**DOI:** 10.1007/s10875-026-01998-z

**Published:** 2026-04-01

**Authors:** Fiona Price-Kuehne, Alice Burleigh, Ying Hong, Ebun Omoyinmi, Gabriela Petrof, Gulshan Malik, Kirsty McLellan, Stephen Owens, Su Han Lum, Pamela Dawson, Inga Turtsevich, Jamie Robertson, Natalie Stolagiewicz, Gareth Roberts, Despina Eleftheriou, Paul Brogan

**Affiliations:** 1 Infection, Immunity and Inflammation Department, University College London Great Ormond Street Institute of Child Health, 30 Guilford Street, London, WC1N 1EH UK; 2 Dermatology Department, Great Ormond Street Hospital for Children NHS Foundation Trust, London, UK; 3 Paediatric Rheumatology Department, Royal Aberdeen Children’s Hospital NHS Grampian, Aberdeen, UK; 4 Paediatric Allergy, Immunology and Infectious Diseases Department, Newcastle Upon Tyne Hospitals NHS Foundation Trust, Newcastle, UK; 5 Paediatric Haematopoietic Stem Cell Transplant Unit, Great North Children’s Hospital, Newcastle upon Tyne, UK; 6 Translational and Clinical Research Institute, Newcastle University, Newcastle upon Tyne, UK; 7 Paediatric Rheumatology Department, Oxford University Hospitals NHS Foundation Trust, Oxford, UK; 8 Rheumatology Department, Frimley Health NHS Foundation Trust, Frimley, UK; 9 Respiratory Medicine Department, Frimley Health NHS Foundation Trust, Frimley, UK; 10 Rheumatology Department, Great Ormond Street Hospital for Children NHS Foundation Trust, London, UK

## Abstract

The sarcoma (Src) family of non-receptor tyrosine kinases (SFKs) regulate innate immunity through their roles in signal transduction and cell migration, adhesion and proliferation. Germline pathogenic variants in this key kinase group are rare but increasingly recognised as causes of autoinflammatory disease. We identified three unrelated kindreds with SFK-associated vasculitis. In Family A, we report a child with systemic vasculitis due to a nonsense heterozygous variant in *HCK* (p.Y515X) who was subsequently treated with allogeneic haematopoietic stem cell transplantation (allo-HSCT), the first use of this approach in SFK-related disease. In Family B, we identified a novel *HCK* missense variant (p.Y522F) causing a cutaneous-limited phenotype. In Family C, we describe a large pedigree carrying a novel missense variant in *FGR* (p.Y523H), establishing *FGR* as a new cause of monogenic vasculitis. In all families, disease-onset was neonatal with vasculitic rash, variably progressing to systemic involvement including lung disease. Functional studies demonstrated reduced Hck and Fgr protein expression and enhanced p-STAT1 and p-STAT5 signalling in monocytes and lymphocytes. With striking similarities in the clinical phenotypes and underlying molecular mechanisms, these cases expand the spectrum of sarcoma (Src) family of non-receptor tyrosine kinase-associated autoinflammatory disease, and support this as a distinct group that we propose is termed the “autoinflammatory Src-opathies (sarcopathies)”.

**Supplementary Information:**

The online version contains supplementary material available at 10.1007/s10875-026-01998-z.

## Introduction

**Fig. 1:**
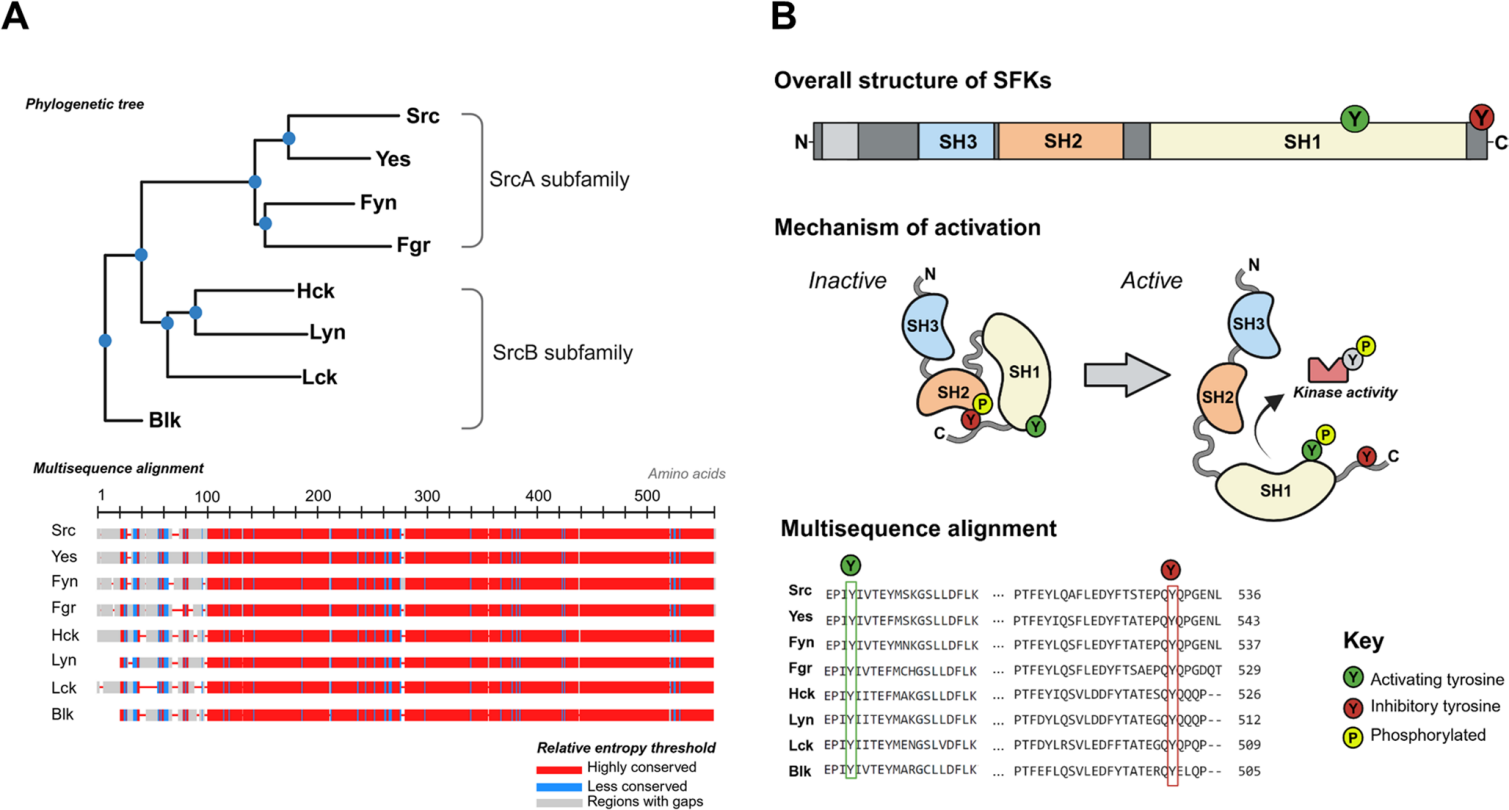
Src-family kinases (SFKs): SrcA and SrcB subfamilies and mechanism of action. (**A**) Phylogenetic tree showing evolutionary segregation of SrcA (Src, Yes, Fyn, Fgr) and SrcB (Hck, Lyn, Lck, Blk) subfamilies, and multi-sequence alignment of SFK protein sequences generated using NCBI Constraint-based Multiple Alignment Tool [18], depicting high sequence homology between subfamily members. (**B**) All SFKs contain distinct structural regions: an N-terminal domain (membrane localisation), SH3 and SH2 domains (protein–protein interactions), the SH1 domain (kinase catalytic domain), and a C-terminal tail (inhibitory regulation). SFK activity is tightly regulated through post-translational modifications of two highly conserved tyrosine residues: phosphorylation of the inhibitory tyrosine in the C-terminal tail maintains the SFK in its closed, autoinhibited conformation, while dephosphorylation of the C-terminal inhibitory tyrosine and phosphorylation of the activating tyrosine in the SH1 kinase domain leads to an open formation and kinase activity. ***Abbreviations***: *Blk*,* B-lymphocyte kinase; Fgr*,* Gardner-Rasheed feline sarcoma viral oncogene homolog; Hck*,* haematopoietic cell kinase; Lck*,* lymphocyte-specific protein tyrosine kinase; Lyn*,* Lck/Yes-related novel tyrosine kinase; SH1*,* Src homology domain 1; SH2*,* Src homology domain 2; SH3*,* Src homology domain 3; SFK*,* Src-family kinase; Yes*,* tyrosine-protein kinase Yes*

Src-family kinases (SFKs) are non-receptor protein tyrosine kinases with key roles in innate immune regulation. So-called due to the discovery of *SRC* as the first proto-oncogene in sarcoma [1], eight SFKs are well-characterised in human. There is high homology across the subfamily members with shared signal transduction pathways, downstream cellular processes and mechanism of activation (Fig. 1). The ubiquitously expressed members (Src, Yes1 and Fyn) are classically implicated in cancer biology, while those with more haematopoietic- and myeloid-restricted expression (Fgr, Hck, Blk, Lck and Lyn) play central roles in immune cell signalling [2–5]. Somatic gain-of-function (GOF) variants in SFKs are well-established as key oncogenic drivers, and importantly, many of the signalling pathways that drive SFK-mediated oncogenesis are also fundamental to immune cell activation and cytokine production [6–8]. This mechanistic link between malignancy and immune dysregulation is reflected in the increasing recognition of germline GOF variants being associated with a haematological or autoinflammatory phenotype. These include *SRC* variants causing autosomal dominant (AD) thrombocytopenia with myelofibrosis [9–12]; *HCK* variants linked to cutaneous and pulmonary vasculitis [13, 14]; *LYN* variants associated with small-vessel vasculitis and liver fibrosis [15, 16]; and an association between rare *FGR* variants and chronic non-bacterial osteomyelitis (CNO) [17] (Supplementary Table 1). Together, these findings suggest that SFK-related diseases belong to a wider group of kinase-driven inflammatory disorders known as kinasopathies.

In this study we analysed whole-exome sequencing (WES) data from families with autoinflammatory disease, two of whom had previously undergone WES without identification of a causal variant. Given the shared biological function of SFKs in innate immune signalling, and phenotypic overlap with *LYN*-associated autoinflammatory vasculitis in the absence of identifiable *LYN* variants, we deliberately prioritised SFK genes for focussed analysis. We identified three cases of *HCK*-associated vasculitis in two unrelated kindreds and describe the first use of allogeneic haematopoietic stem cell transplantation (allo-HSCT) for this recently described disease. Then, in a large pedigree with 13 affected members, we identified a causative novel pathogenic variant in *FGR*. These findings expand the spectrum of SFK-associated autoinflammatory diseases and establish *FGR* as a new cause of monogenic vasculitis.

## Methods

### Study Participants

Ethical approval was obtained from the National Research Ethics Service, Bloomsbury Committee (08/H0713/82). All adult subjects provided written informed consent to participate; parental/legal guardian consent was obtained for all children with age-appropriate consent/assent as required. Specific consent was sought for the use of photographs. Control samples were obtained from adolescent (14–24 years) and adult healthy control subjects with local ethics approval (II/LP/0330).

### Clinical Data

Clinical data were provided by the referring clinician or extracted from archival medical records held at Great Ormond Street Hospital NHS Foundation Trust, London. Relevant human phenotype ontology (HPO) terms were derived for bioinformatic analysis.

### Genetic Testing

Genomic DNA was extracted from peripheral blood (Gentra Puregene blood kit, Qiagen) or saliva (PrepIT L2P kit, DNAGenotek Inc). Whole exome sequencing was performed by Informed Genomics Ltd., and data was analysed as previously published [19]. In brief, variant call format (VCF) files were annotated with ANNOVAR [20] and filtered using an in-house R pipeline incorporating population frequency, a virtual gene panel of 786 genes associated with inflammation/vasculitis, and tools including The Exomiser [21] and ExomeDepth [22]. Variants were additionally reviewed using *in silico* prediction scores including PhyloP (Phylogenetic P-values) to assess evolutionary conservation [23], AlphaMissense, a deep-learning model for pathogenicity prediction based on AlphaFold [24, 25], and REVEL (Rare Exome Variant Ensemble Learner), which integrates multiple tools [26]. Familial co-segregation testing was done by Sanger sequencing, using primers for *HCK* exon 13 (forward: TAATTCCACGGCTCCTTTTCAG; reverse: TCAGGAATTGGAAGGACAGGAA) or *FGR* exon 13 (forward: TAATCCAGCTGTTTCCAGGG; reverse: CTTTGGAAGAACAGCTCTGGG). Products were sequenced by Eurofins Genomics (TubeSeq Supreme), then aligned against the MANE select reference sequences (*HCK*, NM_002110; *FGR*, NM_005248) using CodonCode Aligner.

### Peripheral Blood Mononuclear Cell (PBMC) Isolation

PBMC were isolated from heparinised whole blood by density gradient centrifugation (Lymphoprep™, STEMCELL Technologies), according to manufacturer’s instructions.

### Cell Lysates Preparation and Immunoblotting

PBMC were lysed in RIPA buffer (ThermoFisher Scientific) with 1% protease/phosphatase inhibitor cocktail (Roche), and protein quantified by BCA assay and normalised to 20 µg. Lysates were heated at 95 °C for 5 min with Laemmli buffer (Bio-Rad) and run on a 4–20% SDS-PAGE gel followed by transfer to a polyvinylidene difluoride membrane. Membranes were blocked with 2% bovine serum albumin (BSA) and probed with primary antibodies against Hck (Clone E117F; CST, 14643), Fgr (Clone E3R7L; CST, 2755), or GAPDH (Santa Cruz, sc-47724) for loading control, followed by HRP-conjugated secondary antibodies (Thermo Fisher Scientific). The signal was detected using electrochemiluminescence (PK10013; Proteintech) and images captured with a ChemiDoc Imager (Bio-Rad), then analysed with Image Lab Software (Bio-Rad).

### Measurement of Cytokines and Chemokines

Cytokine and chemokine levels in sera were measured using V-PLEX Proinflammatory Panel Meso Scale Discovery kit (Meso Scale Diagnostics), according to manufacturer’s instructions.

### Quantitative PCR (qPCR) of Interferon Stimulated Genes (ISG)

qPCR was performed by the Camelia Botnar Laboratory, Great Ormond Street Hospital NHS Foundation Trust, as previously described [27, 28]. In brief, blood was collected into PAXgene^®^ tubes (PreAnalytix), and RNA extracted using PAXgene^®^ Blood RNA kit 50 v2 (PreAnalytix). Single-stranded cDNA was generated using High-Capacity cDNA reverse transcription kit (Applied Biosystems). qPCR was then performed using iTaq Universal SYBR Green Supermix (Bio-Rad) and the relevant QuantiTect Primer Assays (Qiagen). The relative abundance of 11 targets (*CXCL10*, *CXCL9*, *IFI27*, *IFI44L*, *IFIT1*, *IFNB1*, *IFNG*, *IFNL1*, *IL18*, *RSAD2*, *SIGLEC1*) was normalised to the expression level of *ACTB* and assessed using CFX Manager software (Bio-Rad).

### STAT Phosphorylation Assays

PBMC were incubated with live/dead fixable violet dead cell stain kit (Invitrogen, L34955) then left untreated or stimulated with 100 ng/ml IFN-α2b (GenScript, 203002-50) for 30 min. Following fixing (Phosflow Fix Buffer, BD Biosciences, 557870) and permeabilisation (Perm Buffer III, BD Biosciences, 558050), cells were stained with PE anti-p-STAT5 (Tyr694) and Alexa-Fluor 488 anti-p-STAT1 (Tyr701) (BD Bioscience 562077 and 612596). Stained cells were acquired on a CytoFLEX flow cytometer (Beckman Coulter Life Sciences). Data analysis, including the delineation of cell populations based on forward and side scatter characteristics, was performed using FlowJo software (BD Life Sciences).

### Integrin Surface Staining

Whole blood was freshly collected in heparinised tubes, aliquoted and incubated for 30 min at 37 °C with 100 ng/ml lipopolysaccharide (LPS, Sigma-Aldrich, L-9764) then diluted in phosphate buffered saline (PBS), or PBS alone for vehicular control, and incubated for 30 min on ice with 1:20 antibodies: PE-CD11b ICRF44 (Biolegend, 982606) and APC-CD18 IB4 (Biolegend, 373406). Following staining, red cell lysis solution was added and the mixture was incubated for 15 min at room temperature then washed and analysed by flow cytometry as above.

### Statistical Analysis and Figures

Due to the limited sample size, findings are presented descriptively. Graphs were produced using Prism Version 9 (GraphPad Software). Figures were prepared using BioRender.

### Data Availability

The novel *HCK* and *FGR* pathogenic variants are submitted to ClinVar (https://www.ncbi.nlm.nih.gov/clinvar/) and the Infevers database (https://infevers.umai-montpellier.fr/web/). Further data is available upon reasonable request to the corresponding author.

## Results

### Clinical Presentations

Results of clinical laboratory, histopathological and imaging investigations are presented in full in Supplementary Tables 2–4.

#### Family A

**Fig. 2:**
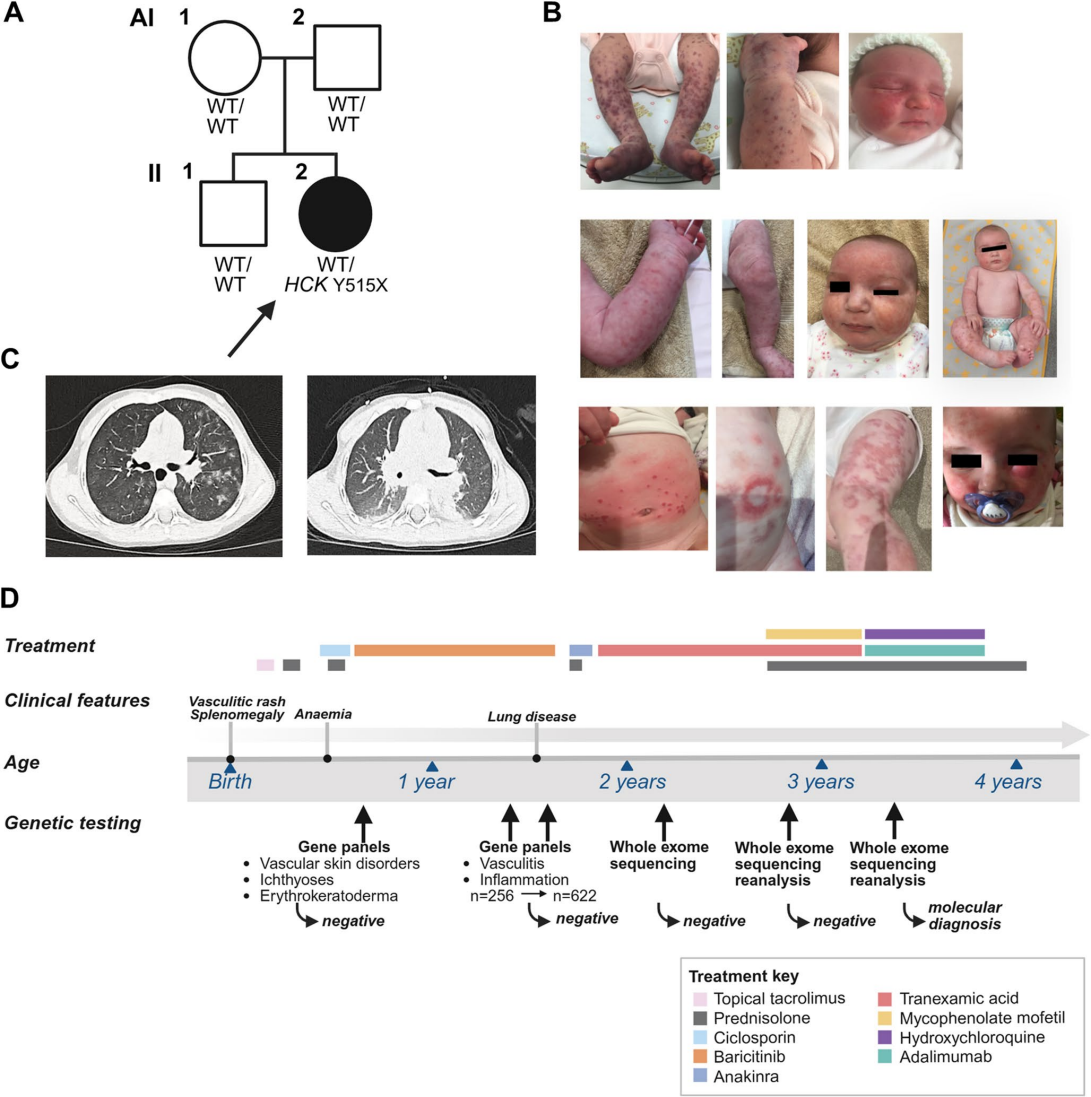
Pedigree and clinical features - Family A. (**A**) Pedigree of Family A with affected individual shaded black, proband indicated with an arrow, and genotype indicated as wild-type (WT/WT) or heterozygote (WT/*HCK* p.Y515X); (**B**) Photographs of vasculitic rash in AII-2 at birth (top row), seven weeks (second row) and seven months (third row) of age; (**C**) CT chest demonstrating pulmonary haemorrhage and hilar lymphadenopathy; (**D**) Timeline from birth to four years, depicting age at onset of clinical features, trials of treatment, and the multiple genetic testing attempts that failed to identify a cause

Proband A (AII-2) from Family A (Fig. 2A) presented at four hours of life with widespread purpuric rash. Blood tests were unremarkable except for a markedly raised D-dimer; splenomegaly was also noted, but bone marrow was normal. Skin biopsy showed leukocytoclastic vasculitis and the rash persisted throughout infancy, waxing and waning but never fully resolving (Fig. 2B). She subsequently developed epistaxis, haemolacria and blood-stained stools leading to iron-deficiency anaemia requiring transfusion, and at 12 months she had recurrence of splenomegaly. From 18 months of age, she developed chronic cough and haemoptysis; chest computed tomography (CT) scan demonstrated enlarged hilar lymph nodes and bilateral inflammatory changes and pulmonary haemorrhage (Fig. 2C). Autoimmune screen and inflammatory markers were repeatedly normal throughout, and there was no clinical response to multiple anti-inflammatory/immunosuppressive therapies including trials of prednisolone, ciclosporin, baricitinib, anakinra, mycophenolate mofetil, hydroxychloroquine and adalimumab. Repeat genetic testing throughout this period also failed to identify a cause (Fig. 2D).

#### Family B

**Fig. 3:**
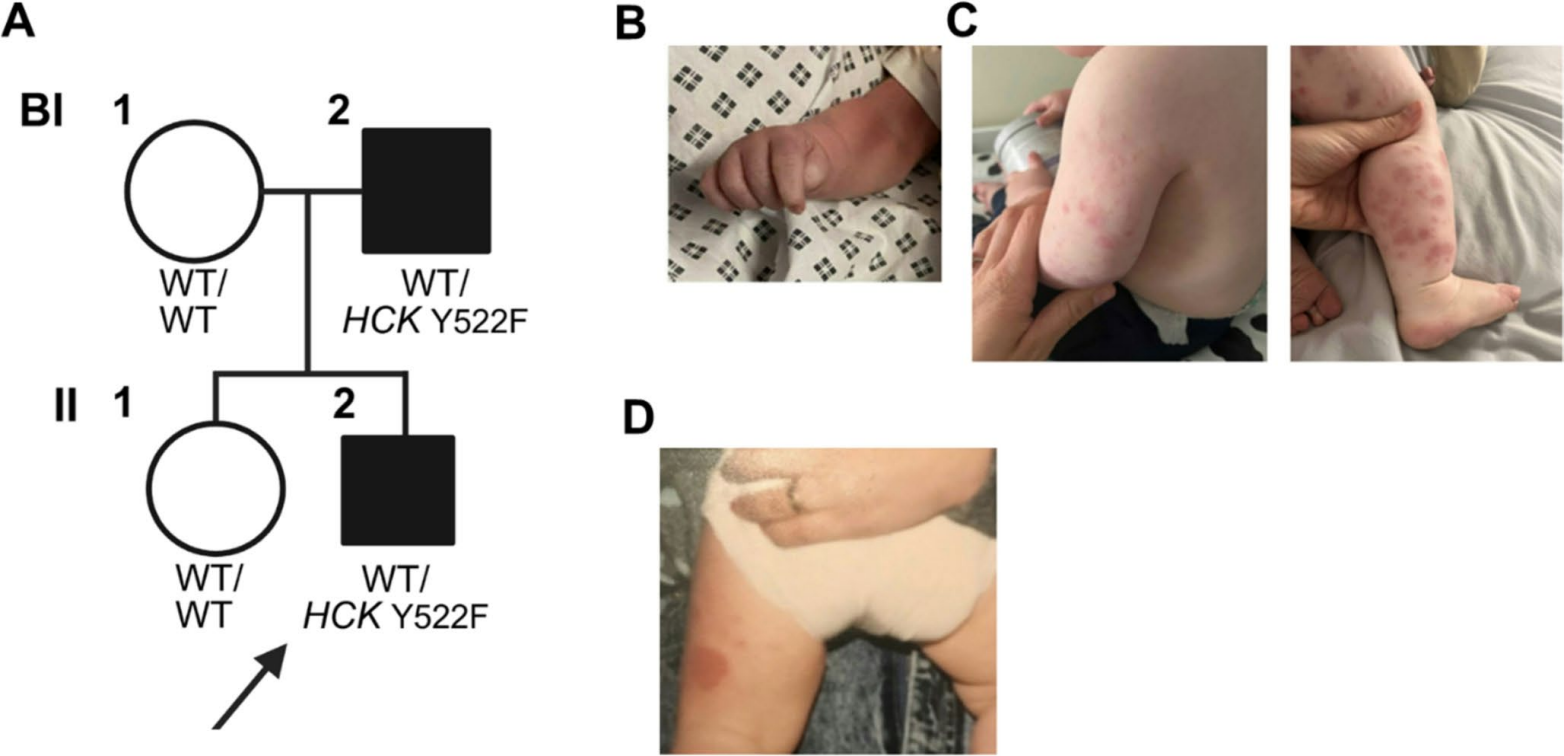
Pedigree and clinical features - Family B. **A**) Pedigree of Family B with affected individuals shaded black, proband indicated with an arrow, and genotype indicated as wild-type (WT/WT) or heterozygote (WT/*HCK* p.Y522F); **B **+** C**) Photographs of vasculitic rash in BII-2 at birth and during the first year of life; and **D**) Rash in BI-2 (family photograph taken during infancy)

Proband B (BII-2) from Family B (Fig. 3A) presented at four hours of life with purpuric rash, predominantly affecting the limbs (Fig. 3B). During infancy, he continued to have episodes of rash, typically triggered by vaccination or intercurrent illness (Fig. 3C). Skin biopsy showed leukocytoclastic vasculitis. Autoimmune serologies were negative, and he had a normal CT chest. His father (BI-2) also had neonatal-onset leukocytoclastic vasculitis (Fig. 3D) which resolved in childhood, and he remains healthy in adulthood.

#### Family C

**Fig. 4:**
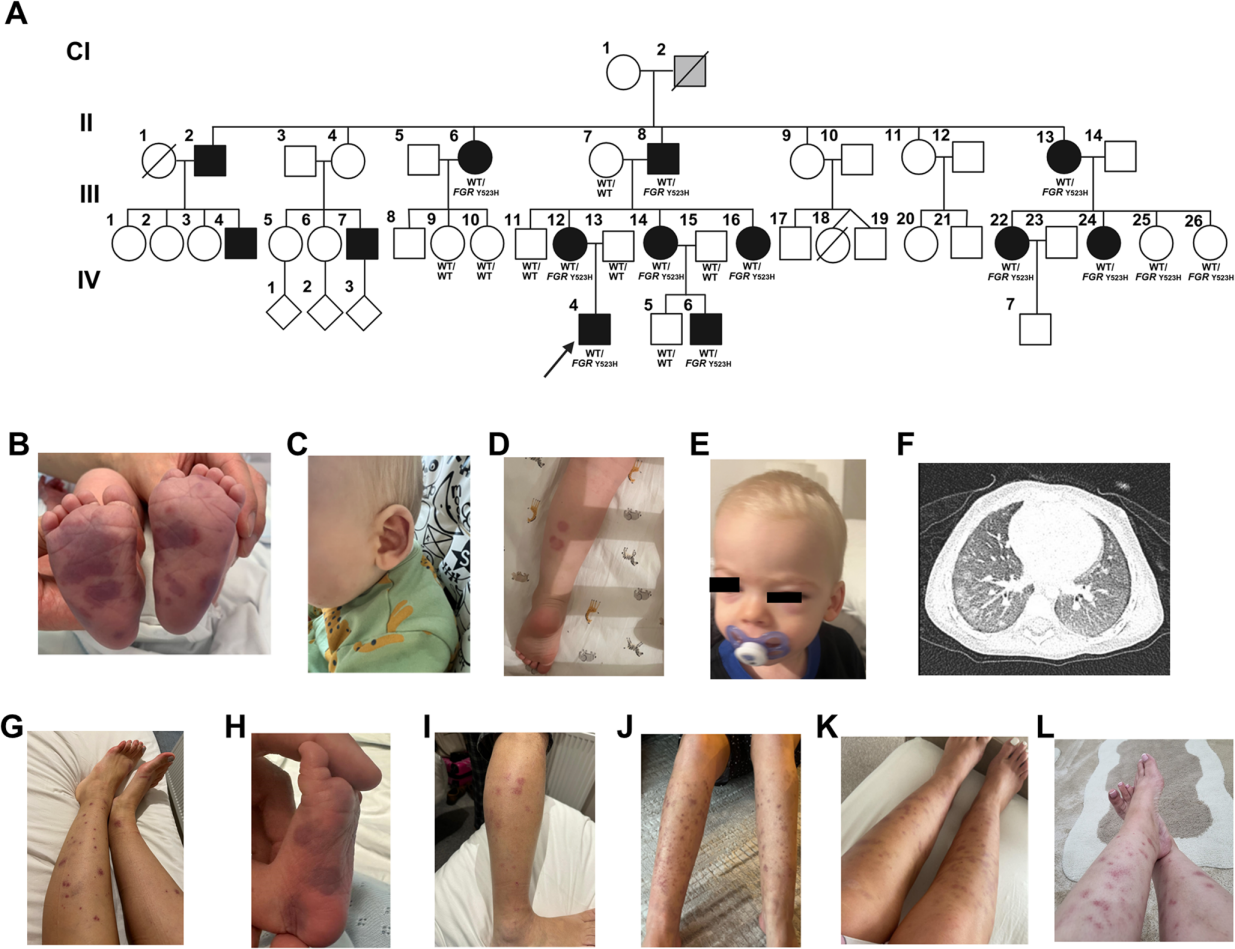
Pedigree and clinical features - Family C. (**A**) Pedigree of Family C with affected individuals shaded black (grey-shaded CI-2 presumed to be affected but deceased and unable to confirm), proband indicated with an arrow, and genotype where available indicated as wild-type (WT/WT) or heterozygote (WT/*FGR* p.Y523H); (**B**) Vasculitic rash in proband CIV-4 at two days of life; (**C**) A further episode with auricular swelling during infancy; and **D **+** E**) at two years of age associated with periorbital swelling; **F**) CT scan at three years of age showing pulmonary haemorrhage. **G**-**L**) Vasculitic rashes in other members of the family: G) CIII-12; H) CIV-6); I) CII-8; J) CIII-22; K) CIII-24; and L) CII-6

Family C comprised 13 affected members across four generations (Fig. 4A). The proband (CIV-4) developed a purpuric rash on day two of life (Fig. 4B) that recurred throughout infancy, sometimes associated with periorbital oedema and auricular swelling (Fig. 4C-E). A diagnosis of hypocomplementaemic urticarial vasculitis was given at the time and a case report published [29], prompting the lead author of our study (FPK) to contact the authors due to the similarity in phenotype to the other cases in our report. It became apparent that repeat complement levels were consistently normal, casting doubt on this initial diagnosis. Then, aged two years he was found to be severely anaemic during an upper respiratory tract infection. Anaemia persisted despite iron supplementation, and CT scan of the chest at three years of age revealed pulmonary haemorrhage and vasculitis (Fig. 4F).

The proband’s mother (CIII-12) had recurrent vasculitic rash from infancy, associated with fatigue and leg pain (Fig. 4G). One of her siblings (CIII-16) was also affected, and additionally was diagnosed with CNO aged 9 years, while another sibling (CIII-14) had milder disease resolving in childhood. The latter’s son (CIV-6) also presented with florid purpuric rash on day 2 of life, predominantly affecting the feet (Fig. 4H).

The proband’s maternal grandfather (CII-8) first presented in infancy with fever, pulmonary haemorrhage and anaemia, diagnosed at the time as idiopathic pulmonary haemosiderosis. At age 32 years, pulmonary changes were interpreted as sarcoidosis. At age 53 years with ongoing respiratory symptoms, he was diagnosed with early-stage mucinous adenocarcinoma for which he underwent lobectomy, but subsequent cancer surveillance imaging showed non-malignant pulmonary nodularity of unknown cause. He also suffered from episodes of vasculitic rash (Fig. 4I).

Additional affected relatives in Family C included three of CII-8’s siblings: CII-13 and two of her two daughters (CIII-22, CIII-24) had neonatal-onset rash persisting into adulthood (Fig. 4J–K), with CIII-22 also having an incidental finding of bilateral lung nodularity on imaging at 18 years of age, then developing episodes of haemoptysis from 20 years of age and recurrence of presumed lung vasculitis of unknown cause, treated with immunosuppression. The proband’s great-aunt (CII-6) also had persistent rash since birth with iron-deficiency anaemia, arthritis and chronic sinusitis in adulthood (Fig. 4L).

### Genetic Investigation Results

WES was performed in probands (AII-2, BII-2, CIV-4) and their affected parents (BI-2, CIII-12) and additional co-segregation testing was performed using Sanger sequencing.

#### Family A

In Family A, AII-2 was heterozygous for a *de novo* variant in *HCK*: NM_002110: c.1545 C > A, p.(Tyr515Ter) [*HCK* p.Y515X].This variant is a rare nonsense variant, not described in population databases, and has been previously reported in a case of neonatal-onset vasculitis [14].

#### Family B

In Family B, BII-2 and his affected father BI-2 had a novel heterozygous missense pathogenic variant in *HCK* in which the inhibitory tyrosine is replaced with phenylalanine: *HCK* (NM_002110): c.1565 A > T (p.Tyr522Phe) [*HCK* p.Y522F]. This novel variant is predicted to be pathogenic by multiple *in silico* tools including PhyloP score = 9.323 (strong evolutionary conservation; values > 2), REVEL score = 0.720 (supportive of pathogenicity; values > 0.5) and AlphaMissense score = 0.7852 (likely pathogenic; values ≥ 0.56).

#### Family C

In Family C, WES of CIV-4 and CIII-12 identified a novel heterozygous variant in *FGR* in which the inhibitory tyrosine is replaced with histidine: *FGR* (NM_005248.3): c.1567T > C, p.(Tyr523His) [*FGR* p.Y523H]. Subsequent Sanger sequencing in further members of Family C confirmed that all clinically affected individuals (*n* = 10) were heterozygous (WT/*F*GR p.Y523H), while four unaffected members were wild-type (WT/WT). Two self-reported healthy members (CIII-25 and CIII-26) were also heterozygous (WT/*FGR* p.Y523H), suggesting AD inheritance with incomplete penetrance or variable expressivity, although these two carriers have not undergone investigations for subclinical disease. Segregation analysis in Family C using the Jarvik-Browning model of informative meioses gave a probability 0.00049 for the observed genotype-phenotype pattern, constituting further strong evidence for pathogenicity (Supplementary Fig. 1) [30]. The novel *FGR* p.Y523H pathogenic variant is also predicted to be pathogenic by multiple *in silico* tools including PhyloP score = 9.268 (highly conserved), REVEL score = 0.717 (supportive of pathogenicity) and AlphaMissense score = 0.9828 (likely pathogenic).

### Functional Characterisation in Cells From Affected Individuals

#### Hck Protein Expression in AII-2 and BII-2

**Fig. 5:**
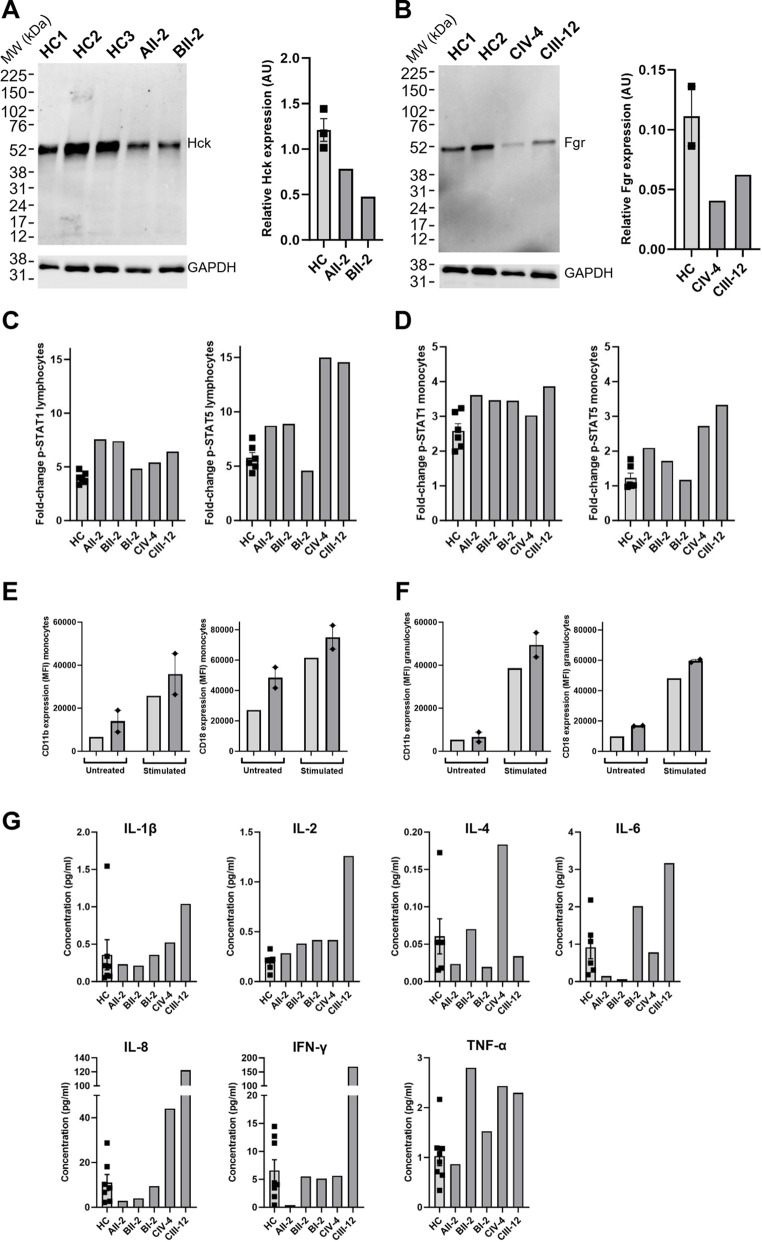
Functional characterisation of immune cells from affected individuals: immunoblotting, JAK–STAT signalling, integrin expression, and cytokine profiling. (**A**) Immunoblot and relative quantification of total Hck in PBMC lysates from AII-2 and BII-2 compared to HC; (**B**) Immunoblot and relative quantification of total Fgr in PBMC lysates from CIII-12 and CIV-4 compared to HC; **C **+** D**) Fold-change (stimulated vs. baseline) in MFI in p-STAT1 and p-STAT5 levels in lymphocytes and monocytes following stimulation with IFN-α2b; **E **+** F**) CD11b and CD18 surface expression on untreated and stimulated monocytes and granulocytes in CIII-12 and CIV-4; **G)** Cytokine and chemokine concentrations in serum. Data points represent biological replicates; error bars show mean ± SEM. ***Abbreviations***: *AU*,* arbitrary units; CD11b*,* cluster of differentiation molecule 11b; CD18*,* cluster of differentiation molecule 18; FGR*,* Gardner-Rasheed feline sarcoma viral oncogene homolog; GAPDH*,* glyceraldehyde 3-phosphate dehydrogenase; HC*,* healthy control; HCK*,* haematopoietic cell kinase; IFN-γ*,* interferon gamma; IL*,* interleukin; MFI*,* median fluorescence intensity; PBMC*,* peripheral blood mononuclear cell; pg*,* picograms; p-STAT*,* phosphorylated signal transducer and activator of transcription; TNF-α*,* tumour necrosis factor alpha; WT*,* wild type*

To examine whether the *HCK* p.Y515X and *HCK* p.Y522F variants altered Hck protein expression, we examined Western blotting of PBMC lysates, and this revealed reduced Hck protein in AII-2 (WT/*HCK* p.Y515X) and BII-2 (WT/*HCK* p.Y522F) compared to healthy controls (HC) (Fig. 5A). Median relative Hck expression in affected cases was approximately half that of HC, consistent with heterozygous loss.

#### Fgr Protein Expression in CIV-4 and CIII-12

To examine the effect of the *FGR* p.Y523H variant on Fgr protein expression, we examined Western blotting of PBMC lysates, and this revealed reduced Fgr protein in CIV-4 and CIII-12 (WT/*FGR* p.Y523H) compared to HC (Fig. 5B). Median relative Fgr expression in heterozygotes was again approximately half that of HC, consistent with heterozygous loss.

#### p-STAT1 and p-STAT5 Response To IFN-α2b in AII-2, BII-2, BI-2, CIII-12 and CIV-4

In view of previous studies that have shown the ability of Hck to activate downstream STAT pathways, we then examined STAT signalling in cells derived from AII-2, BII-2, BI-2, CIII-12 and CIV-4 by measuring intracellular p-STAT1 and p-STAT5 in PBMC at baseline and following stimulation with IFN-α2b.The fold-change (stimulated vs. baseline) expression of p-STAT1 and p-STAT5 in lymphocytes and monocytes measured in median fluorescence intensity (MFI) was modestly increased in all probands and symptomatic individuals (AII-2, BII-2, CIV-4, CIII-12) compared with HC, although the magnitude of change varied between individuals and was less consistent in monocytes. In contrast, BI-2 (now asymptomatic in adulthood) showed fold-change levels similar to HC (Fig. 5C-D).

#### Surface Expression of CD11b/CD18 in CIII-12 and CIV-4

CD11b and CD18 form the integrin Mac-1, a key regulator of adhesion and immune cell activation, and is known to have a key role in Src-related pathology [31, 32]. Therefore, we next examined CD11b and CD18 expression to assess whether the *HCK* variants impacted downstream myeloid cell function. The integrin signalling pathway was assessed in CIII-12 and CIV-4 by measuring surface expression of CD11b and CD18, at baseline and in response to stimulation with LPS. There was slightly increased expression of CD11b and CD18 in monocytes and granulocytes, measured as MFI, both at baseline and in response to LPS compared to HC (Fig. 5E-F). Assays including Families A and B were not possible due to limited sample availability.

#### Cytokine and Chemokine Levels in Serum From AII-2, BII-2, CIII-12 and CIV-4

To examine whether the variants induced pro-inflammatory cytokine and chemokine release, serum cytokine and chemokine concentrations were measured next. Levels varied between affected cases. AII-2 had levels largely comparable to HC across most cytokines; BII-2 showed modest increases in IL-2, IL-8, IFN-γ and TNF-α; and CIV-4 and CIII-12 had more marked elevations, particularly in IL-6, IL-8, IFN-γ and TNF-α, with smaller increases in IL-1β, IL-2 and IL-4. However, no consistent pattern was observed across all cases (Fig. 5G).

#### Interferon-Stimulated Gene (ISG) Expression in AII-2 and BII-2

Given that SFKs can modulate IFN-induced immune pathways, we also assessed ISG expression in AII-2 and BII-2. ISG expression was within normal range in both probands from Family A and Family B (Supplementary Table 4).

### Use of Allogeneic Haematopoietic Stem Cell Transplant (Allo-HSCT) in AII-2

The only previously reported case of *HCK*-associated vasculitis (with the identical *HCK* p.Y515X variant) had died in adolescence of respiratory failure [14]. In keeping with this severe phenotype, patient AII-2 demonstrated a highly refractory disease course, and failed multiple systemic immunomodulatory therapies (Fig. 2). Why this disease is so refractory is mechanistically unknown, but likely reflects pathological loss of inhibitory function of the Hck protein with impact on multiple downstream innate immune signalling pathways. In light of the severity of her disease and the fatal outcome reported previously, aged four years, AII-2 underwent a fully matched unrelated donor allo-HSCT following reduced-toxicity myeloablative conditioning and an intensification of steroid treatment. Her transplant course was uncomplicated, with platelet engraftment on day + 11 and neutrophil engraftment on day + 13. By four months post-transplant, her rash had resolved completely for the first time since birth, chest CT had normalised, and respiratory symptoms had resolved. At six months, she remained well with donor chimerism > 95% and without any immunosuppressive and/or anti-inflammatory medication.

## Discussion

In this report, we describe heterozygous pathogenic variants in SFK genes in three unrelated kindreds: a previously described *de novo HCK* nonsense pathogenic variant in Family A (*HCK* p.Y515X); a novel *HCK* AD-inherited missense pathogenic variant in Family B (*HCK* p.Y522F); and a novel *FGR* AD-inherited missense pathogenic variant in Family C (*FGR* p.Y523H). These findings expand the spectrum of SFK-associated autoinflammatory diseases and establish *FGR* as a new cause of monogenic vasculitis.

A theme of this study was the value of reanalysis of genomic data. It is now well-recognised that repeated analyses over time of WES data provides increased diagnostic yield [33]. The pathogenic *HCK* variants in Families A and B were not identified on earlier WES because our initial pipelines prioritised known disease-associated genes, which in the case of *HCK* had not yet been published. This emphasises the importance of reanalysis of data over time for unsolved cases. According to the American College of Medical Genetics and Genomics (ACMG) variant classification criteria [34], the two *HCK* variants (p.Y515X and p.Y522F) met criteria for Pathogenic classification, supported by functional evidence, localisation to the conserved C-terminal regulatory tail, absence from population databases, and additional computational and inheritance-based evidence. In contrast, the *FGR* p.Y523H variant met criteria for Likely Pathogenic, based on functional and computational evidence and localisation to the same critical regulatory region, but with more limited segregation and gene-level evidence reflecting its status as a novel disease-gene in a single family. This highlights a recognised limitation of the ACMG framework, which is conservative and can be difficult to apply in ultra-rare, newly described disease contexts.

All probands presented in the neonatal period with a vasculitic rash, which continued throughout childhood, often exacerbated by intercurrent illness or vaccination. Disease severity ranged from persistent systemic vasculitis with pulmonary haemorrhage and anaemia (Family A), to a milder cutaneous-limited course (Family B), and a variably expressed skin and lung vasculitis (Family C). In Family C, incomplete penetrance was suggested by the presence of heterozygous carriers without overt symptoms, although subclinical or pre-symptomatic disease could not be excluded; and notably, in at least two individuals (CII-8 and CIII-22), lung vasculitis was incidentally found only on screening for other conditions. In AII-2, we also report the first use of allo-HSCT for *HCK*-associated vasculitis. The successful early outcome provides possible evidence that allo-HSCT can be curative for *HCK*-associated vasculitis, although long-term follow-up will be needed. Pharmacological inhibition of SFKs using small-molecule inhibitors such as dasatinib has been shown to suppress downstream signalling pathways, providing a possible means for targeted treatment [35, 36], with dasatinib approved for use in certain leukaemias [37]. However, the long-term use of such agents in the context of autoinflammatory disease remains exploratory, and would require careful evaluation of safety and tolerability. In AII-2, the use of small molecule SFK inhibitors as a bridge to transplant was discussed but decided against due to lack of evidence, and uncertainty about their impact on the stem cell compartment.

**Fig. 6:**
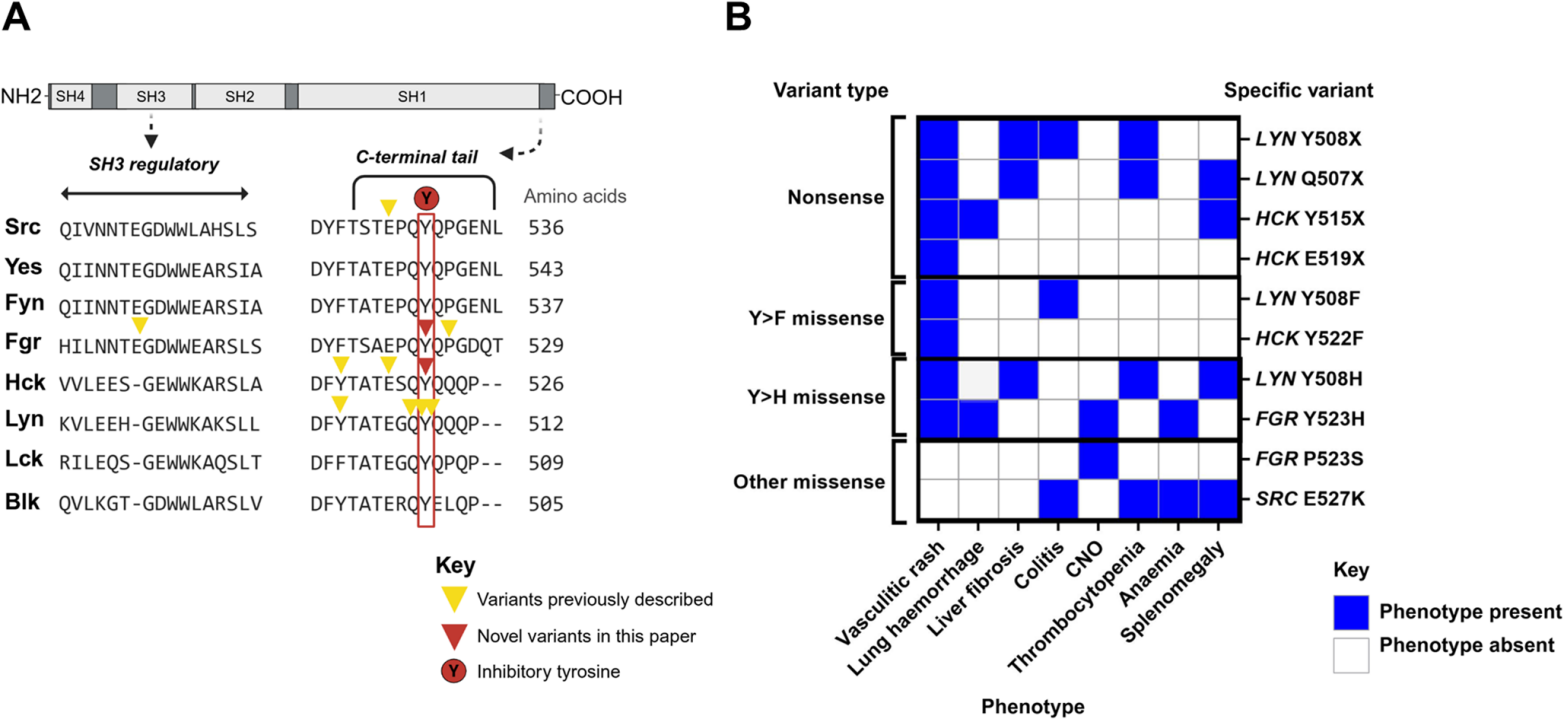
Pathogenic variants in Src-family kinases (SFKs) cluster at the C-terminal tail and share clinical phenotypes. (**A**) Multi-sequence alignment of Src-family kinases (SFKs) with the conserved inhibitory tyrosine in the C-terminal tail (red box) and positions of previously reported germline variants (yellow triangles) and novel variants identified in this study (red triangles) indicated; (**B**) Genotype-phenotype correlation of reported germline gain-of-function SFK pathogenic variants (listed as amino acid p. change), with phenotypes consistently including neonatal-onset vasculitic rash and variably involving lung, liver, gastrointestinal, haematological and systemic manifestations. ***Abbreviations***: *CNO*,* chronic non-bacterial osteomyelitis*

These cases mirror the phenotypic and genotypic features seen in other SFK-related diseases, and of note, 8/9 of the reported germline variants in SFKs to date affect the C-terminal tail (Fig. 6). This site is crucial for autoinhibition of kinase activity, and disruption leads to constitutive kinase activation. Both novel variants we describe are missense variants affecting the inhibitory tyrosine in the C-terminal tail. Although the heterozygous *HCK* p.Y522F variant identified in Family B is novel in humans, it is well-established in mouse models and cell lines as leading to a constitutively active Hck, and homozygous *HCK* p.Y522F mice spontaneously acquire a lung pathology characterised by extensive eosinophilic and mononuclear cell infiltration [38]. In Family C the pathogenic variant consists of a potentially less deleterious amino acid change compared to Family B since unlike phenylalanine, histidine is phosphorylatable. However, histidine is unique in being the only phospho-amino acid that has two isoforms depending on where the phosphate is attached, and thus it is reasonable to speculate on possible differences in function/stability depending on the phosphorylation site [39]. As per the original *HCK* report [14] the variants therefore likely lead to loss of normal physiological inhibitory function of Hck and we suspect that the same is true for Fgr [17].

In both *HCK*- and *FGR*-mutant individuals, we observed reduced protein levels of Hck and Fgr, respectively, as previously described in *HCK*-associated vasculitis [14]. Since all pathogenic variants occur in the final exon and are predicted to escape nonsense-mediated decay, the mechanism is in keeping with increased degradation of the mutant protein rather than haploinsufficiency. Downstream SFK signalling also appeared to be exaggerated: symptomatic individuals (AII-2, BII-2, CIV-4 and CIII-12) showed enhanced STAT1/STAT5 phosphorylation in response to IFN-α2b, while CIV-4 and CIII-12 were additionally found to have increased β2 integrin (CD11b/CD18) expression following LPS stimulation. These data suggest that loss of the inhibitory tyrosine likely leads to constitutive or dysregulated kinase activity and amplified immune signalling.

Our study has some limitations. Firstly, the number of affected individuals was small, reflecting the rarity of germline SFK variants. Secondly, functional analyses were constrained by limited access to patient-derived material. In particular, we were unable to directly measure kinase activity, and functional studies were performed in primary cells rather than in stably expressing model systems. Thirdly, healthy control samples were obtained from adolescents or adults that were age-similar to the majority of the subjects in the pedigree, but older than the paediatric probands in the studied families. Future studies incorporating model systems, direct kinase assays, and longitudinal sampling will be required as additional cases are identified to fully delineate the molecular mechanisms and clinical spectrum of SFK-associated autoinflammatory disease. Our study was not designed to determine biomarkers of disease severity. Enhanced downstream signalling raises the possibility that these functional readouts could serve as biomarkers of disease activity or treatment responses, but the cross-sectional nature of the analyses precluded assessment of longitudinal changes in signalling in relation to disease activity or treatment.

In Family C, we observed two self-reported healthy individuals carrying the pathogenic *FGR* p.Y523H pathogenic variant. Large pedigrees such as this one are exceedingly rare. In small kindreds with *de novo* monogenic autoinflammatory disease, there may be assumption of full penetrance. However, over time, as these children live to reproductive age and larger pedigrees become available, variable expressivity may become more commonly observed. In fact, the prevalence of incomplete penetrance and variable expressivity is estimated to be over 20% in inborn errors of immunity, and this itself is likely an underestimate due to bias in non-reporting of families with imperfect genotype-phenotype segregation, and an increasing recognition of the contribution of autosomal random monoallelic expression (ARMAE) [40, 41].

In conclusion, we have expanded the clinical and genetic spectrum of SFK-associated autoinflammation and described pathogenic variants in *FGR* as a new cause of monogenic vasculitis. Together, these findings support the recognition of an emerging subgroup of diseases we propose is termed the “autoinflammatory Src-opathies (sarcopathies)”.

## Supplementary Information

Below is the link to the electronic supplementary material.

Supplementary Material 1 (DOCX 530 KB)

## Data Availability

The novel *HCK *and *FGR *pathogenic variants are submitted to ClinVar (https://www.ncbi.nlm.nih.gov/clinvar) and the Infevers database (https://infevers.umai-montpellier.fr/web). Further data is available upon reasonable request to the corresponding author.
