# Supplementary material for: Expanding the Spectrum of Src-Family Kinase-Related Autoinflammatory Diseases: Monogenic Vasculitis Caused By Germline Pathogenic Variants in HCK and FGR
Source: J Clin Immunol. 2026 Apr 1;46(1):51. doi: 10.1007/s10875-026-01998-z (PMC13167823; doi:10.1007/s10875-026-01998-z)

**Supplementary Table 1**: Published cases of human disease caused by germline gain-of-function pathogenic variants in genes encoding for SRC-family of non-receptor tyrosine kinases (SFKs)

**Supplementary Table 2**: Clinical summaries of affected members of Family A and B

**Supplementary Table 3**: Clinical summaries of affected members of Family C

**Supplementary Table 4**: Genotypes and laboratory findings of affected members of Families A-C

**Supplementary Figure 1**: Co-segregation testing and Sanger chromatograms

| **Supplementary Table 1: Published cases of human disease caused by germline gain-of-function variants in genes encoding for SRC-family of non-receptor tyrosine kinases (SFKs)** | | | | | | | | | | | | |
| --- | --- | --- | --- | --- | --- | --- | --- | --- | --- | --- | --- | --- |
| **Gene (transcript)** | ***LYN*** (NM_002350) | | | | ***HCK*** (NM_002110) | | ***FGR*** (NM_005248) | | ***SRC*** (NM_198291) | | | |
| **Number of cases (families)** | 1 case | 1 case | 1 case | 1 case | 1 case | 5 cases  (1 family) | 1 case | 1 case | 9 cases  (1 family) | 7 cases  (1 family) | 1 case | 1 case |
| **Variant details** | | | | | | | | | | | | |
| **Exon** | 13 | 13 | 13 | 13 | 13 | 13 | 5 | 13 | 14 | | | |
| **DNA variant** | c.C1524G | c.A1523T | c.C1519T | c.T1522C | c.C1545A | c.G1555T | Not stated | Not stated | c.G1579A | | | |
| **Amino acid change** | p.Y508X | p.Y508F | p.Q507X | p.Y508H | p.Y515X | p.E519X | p.R118W | p.P525S | p.E527K | | | |
| **Type** | Nonsense | Missense | Nonsense | Missense | Nonsense | Nonsense | Missense | Missense | Missense | | | |
| **Affected protein domain** | C-terminal tail | C-terminal tail | C-terminal tail | C-terminal tail | C-terminal tail | C-terminal tail | SH2 domain | C-terminal tail | C-terminal tail | | | |
| **Zygosity** | Het | Het | Het | Het | Het | Het | Het | Het | Het | | | |
| **Mode of inheritance** | N/A  (de novo) | N/A  (de novo) | N/A  (de novo) | N/A  (de novo) | N/A  (de novo) | AD with reduced penetrance | N/A  (de novo) | Unknown | AD | AD | N/A  (de Novo) | N/A  (de novo) |
| **Demographics** | | | | | | | | | | | | |
| **Sex** | M | M | M | F | F | M+F | Not stated | Not stated | M+F | M+F | M | F |
| **Age at onset** | 1^st^ day of life | 1^st^ day of life | 1^st^ day of life | 1 month (with extreme preterm birth) | 1^st^ day of life | Neonatal | Not stated | Not stated | Childhood | Neonatal/  childhood | Neonatal | Early childhood |
| **Age at molecular diagnosis** | 2 years 6 months | 15 years | 4 months | 2 years | 13 years | 13 to >51 years | Not stated | Not stated | Not stated | Not stated | 2 years | 5 years |
| **Clinical features** | | | | | | | | | | | | |
| **Presenting symptoms** | Skin rash, hepatomegaly, splenomegaly, fever, thrombo-cytopaenia | Skin rash, fever | Skin rash, hepatomegaly, splenomegaly, thrombo-cytopaenia | Skin rash, fever, arthritis | Skin rash | Skin rash | Chronic non-bacterial osteomyelitis | Chronic non-bacterial osteomyelitis | Thrombo-cytopaenia, myelofibrosis, splenomegaly, bleeding, bone pathologies | Thrombo-cytopaenia, bleeding, epilepsy, development delay | Thrombo-cytopaenia,  bleeding, hemiparesis secondary to cerebral haemorrhage | Thrombo-cytopaenia, easy bruising |
| **Anaemia** | Yes | No | No | No | Yes | No | No | No | No | No | No | No |
| **Lung disease** | No | No | No | Yes (coexisting prematurity related lung disease) | Yes | No | No | No | No | No | No | No |
| **Liver involvement** | Yes – hepatomegaly, fibrosis | No | Yes – hepatomegaly, fibrosis | Yes – hepatomegaly, liver calcification | Yes –  hepatomegaly | No | No | No | No | No | No | No |
| **Spleno-megaly** | Yes | No | Yes | Yes | Yes | No | No | No | Yes | Yes | Yes - mild | No |
| **Other features** | Hydrops fetalis, parotitis, periorbital oedema and erythema, conjunctivitis, epididymitis, headaches, arthralgias, oral ulcers, fatigue, colitis | Recurrent periorbital oedema and erythema, conjunctivitis, epididymitis, headaches, arthralgias, oral ulcers, fatigue, abdominal pain, colitis | IUGR, liver fibrosis, transient periorbital erythema, jaundice | Prematurity attributed to maternal HELLP syndrome and IUGR Cardiomegaly, hepatospleno-megaly, abdominal pain, diarrhoea, arthritis | Anaemia, hepatospleno-megaly, lung haemorrhage leading to chronic post-haemorrhagic interstitial changes and fibrosis | None | No | No | Decreased bone mineral density, fractures, and tooth loss, mild dysmorphism | Variable autoimmune/  immunologic features -psoriasis, inflammatory bowel disease, pericarditis, hypogamma-globulinemia. Neurology - seizures | Anaemia | Autism, joint hyperlaxity, facial dysmorphism |
| **Infections** | Entero-pathogenic E. coli, Salmonella, Toxocara canis | Post-streptococcal glomerulo-nephritis | Late neonatal sepsis, asymptomatic COVID-19 | Staphylo-coccus capitis | Respiratory tract - BK polyomavirus, mycobacteria *xenopi* | None described | None described | None described | None described | Respiratory tract and skin infections - pityriasis versicolor | None described | None described |
| **Treatment** | Poor response to IVIG, IVMP and oral prednisolone. Partial response to dasatinib monotherapy. Partial response to etanercept monotherapy. Good response to dasatinib and etanercept combination | No response to anakinra and tocilizumab. Partial response to colchicine. Good response to combination etanercept and colchicine | Etanercept therapy led to partial improvement, but liver fibrosis persisted | Canakinumab | Partial response to prednisolone, IVIG, cyclophos-phamide, plasma-pheresis, ciclosporin, azathioprine, hydroxy-chloroquine, rituximab, alemtuzumab. Some response to ruxolitinib -died of respiratory disease | No treatment | Not stated | Not stated | Splenectomy in one patient | Variable, including splenectomy, prednisolone, or systemic treatment | Referred for consideration of haemato-poietic stem cell transplant – outcome not stated | Not stated |
| **Article details**  **(PMID)** | De Jesus *et al*., 2023  (PMID: 36932076) | | | Louvrier *et al*., 2023 (PMID: 36122175) | Kanderova *et al*., 2022 (PMID:34536415) | Bronz *et al*., 2019  (PMID: NA) | Abe *et al*., 2019  (PMID: 31138708) | | Turro *et al.*, 2016  (PMID: 26936507) | Palma-Barqueros *et al.*, 2022  (PMID:35349645) | Barozzi *et al.*, 2021  (PMID: 33054137) | De Kock *et al.*, 2019 (PMID:31204551) |

***Abbreviations****: AD, autosomal dominant; COVID-19, Coronavirus disease 2019; CRP, C-reactive protein; DNA, Deoxyribonucleic acid; E. coli, Escherichia coli; F, female; FGR, Feline Gardner-Rasheed; HCK, Hematopoietic cell kinase; HELLP, Haemolysis, elevated liver enzymes and low platelet count; Het, heterozygous; IUGR, Intrauterine growth restriction; IV, intravenous; IVIG, Intravenous immunoglobulin; IVMP, Intravenous methylprednisolone; LYN, Lck/Yes-Related Novel Tyrosine Kinase; M, male; NM, Nucleotide accession number; SH2, Src homology 2 domain; SRC, Src kinase.*

| **Supplementary** **Table 2: Clinical summaries of affected members of Family A and B** | | | |
| --- | --- | --- | --- |
| **Case** | | | |
|  | **AII-2** | **BII-2** | **BI-2** |
| **Demographics** | | | |
| **Sex** | Female | Male | Male |
| **Age at onset** | Day 1 of life | Day 1 of life | Infancy |
| **Current age** | 5 years | 3 years | 38 years |
| **Genotypes** | | | |
| **Variant** | *HCK* p.Y515X | *HCK* p.Y522F | *HCK* p.Y522F |
| **Zygosity** | Heterozygous | Heterozygous | Heterozygous |
| **Clinical features** | | | |
| **Skin rash** | Yes | Yes | Yes |
| **Iron-deficiency anaemia** | Yes | No | Not known |
| **Respiratory symptoms** | Haemoptysis | No | No |
| **Other clinical features** | Epistaxis, haemolacria | Mild speech delay | None reported |
| **Imaging** | | | |
| **Abdominal ultrasound** | Day 3 - splenomegaly 3.6 standard deviations over mean. Repeat at 6 weeks – normal | Normal | ND |
| **Echocardiography** | Normal | ND | ND |
| **Chest CT scan** | Multiple bilateral foci suggestive of pulmonary haemorrhage.  Interval scan at 3 months– ground glass opacities with bilateral bulky hilar lymphadenopathy | Normal – no evidence diffuse parenchymal or interstitial lung disease | ND |
| **Other imaging** | CT head – normal | CT head – normal | ND |
| **Histopathology** | | | |
| **Skin biopsy** | Moderate to severe neutrophil infiltrate within and surrounding small vessel walls, scattered neutrophil polymorphs within the dermal collagen, some extravasation of red blood cells, unremarkable subcutaneous fat, non-specific immunofluorescence staining | Normal epidermis. In the underlying dermis and extending focally into the subcutaneous tissue - mild to focally moderate inflammatory cell infiltrate and mild perivascular inflammation including neutrophils and eosinophils and some nuclear debris. A stage in evolution of leukocytoclastic vasculitis was considered histologically with clinicopathological correlation advised. | Leukocytoclastic vasculitis |
| **Broncho-alveolar lavage** | Negative for bacterial culture and extended virology (prior to allo-HSCT) | ND | ND |
| **Bone marrow examination** | Trephine at birth – normal appearances, no evidence of an abnormal cellular infiltrate, increased blast cell population or morphological abnormality  Trephine and aspirate at 2 years of age - reactive bone marrow with granulocyte hyperplasia and mild erythroid dyshaematopoiesis, no evidence of malignancy or mast cell disorder | ND | ND |
| **Other** | Upper and lower GI biopsy – no abnormality, normal mucosa |  |  |
| **Treatment** | | | |
| **Previous treatment** | Topical tacrolimus  Topical mometasome  Prednisolone  Ciclosporin  Baricitinib  Anakinra  Tranexamic acid  Mycophenolate mofetil  Hydroxychloroquine  Adalimumab | Nil | Nil |
| **Current treatment** | Underwent allo-HSCT aged 4 years | Nil | Nil |

*Abbreviations: CT, computerised tomography; GI, gastrointestinal; HSCT, haematopoietic stem cell transplant; ND, not done.*

| **Supplementary Table 3: Clinical summaries of affected members of Family C** | | | | | | | | | | | |
| --- | --- | --- | --- | --- | --- | --- | --- | --- | --- | --- | --- |
| **Cases** | | | | | | | | | | | |
|  | CII-6 | CII-8 | CII-14 | CIII-12 | CIII-14 | CIII-16 | CIII-22 | CIII-24 | CIV-4 | CIV-6 |  |
| **Demographics** | | | | | | | | | | | |
| **Sex** | Female | Male | Female | Female | Female | Female | Female | Female | Male | Male |  |
| **Age at onset** | Infancy | Infancy | Infancy | Infancy | Infancy | Infancy | Infancy | Infancy | Infancy - Day 2 of life | Infancy - Day 2 of life |  |
| **Current age (years, months)** | 58y, 10m | 57y, 7m | 46y, 7m | 33y | 27y, 6m | 24y, 0m | 23y, 11m | 21y, 9m | 3y, 4m | 1y, 6m |  |
| **Genotypes** | | | | | | | | | | | |
| **Variant** | *FGR* p.Y523H | *FGR* p.Y523H | *FGR* p.Y523H | *FGR* p.Y523H | *FGR* p.Y523H | *FGR* p.Y523H | *FGR* p.Y523H | *FGR* p.Y523H | *FGR* p.Y523H | *FGR* p.Y523H |  |
| **Zygosity** | Heterozygous | Heterozygous | Heterozygous | Heterozygous | Heterozygous | Heterozygous | Heterozygous | Heterozygous | Heterozygous | Heterozygous |  |
| **Clinical features** | | | | | | | | | | | |
| **Skin rash** | Yes | Yes | Yes | Yes | Yes | Yes | Yes | Yes | Yes | Yes |  |
| **Iron-deficiency anaemia** | Yes - childhood | Yes - childhood | Yes - childhood | Yes - childhood | UN | UN | Yes | UN | Yes | UN |  |
| **Respiratory symptoms** | Exertional dyspnoea  Chest pain | Exertional dyspnoea  Dry cough | Nil | Nil | Nil | Nil | Haemoptysis  Dyspnoea  Reduced exercise tolerance | Nil | Nil | Nil |  |
| **Other clinical features** | Recurrent sinusitis treated with antibiotics and lavage  Fatigue  Leg pain  Ankle swelling | Arthritis hands/knees  Fatigue  Hypertension  Lung adenocarcinoma (age 53y) | Nil | Fatigue  Leg pain | Nil | Chronic non-bacterial osteomyelitis  Fatigue  Leg pain | Fatigue  Leg pain | Fatigue  Leg pain | Periorbital swelling  Ear erythema  Autism spectrum disorder | Nil |  |
| **Lung function tests** | ND | Moderate restrictive pattern | ND | ND | ND | ND | Reduced DLCO | ND | ND | ND |  |
| **Imaging** | | | | | | | | | | | |
| **Abdominal ultrasound** | Normal | ND | ND | ND | ND | ND | ND | ND | ND | ND |  |
| **Chest X-ray** | Normal | Bilateral nodularity | ND | ND | ND | ND | Bilateral nodularity | ND | Normal | ND |  |
| **Chest CT scan/ other imaging** | Chest CT – normal.  CT sinus – moderate mucosal thickening.  CTPA – normal  CT coronary angiogram – normal. | Chest CT - Extensive peribroncho-vascular and perilymphatic nodularity throughout both lungs. | ND | ND | ND | ND | Chest CT - Bilateral ground-glass changes, pulmonary haemorrhage. | ND | Chest CT - Bilateral ground-glass changes, pulmonary haemorrhage. | ND |  |
| **Histopathology** | | | | | | | | | | | |
| **Lung biopsy** | ND | Lung lymph node – no granuloma/  malignancy | ND | ND | ND | ND | ND | ND | ND | ND |  |
| **Broncho-alveolar lavage** | ND | Haemosiderin-laden macrophages | ND | ND | ND | ND | Haemosiderin-laden macrophages | ND | ND | ND |  |
| **Treatment** | | | | | | | | | | | |
| **Previous treatment** | Antibiotics for sinusitis | Treated for sarcoidosis – details unknown.  Lobectomy for lung cancer | Nil | Nil | Nil | Methotrexate | Prednisolone  Azathioprine  Cyclophos-phamide | Nil | Blood transfusions and iron supplements | Nil |  |
| **Current treatment** | Saline sinus/nasal rinse | Bronchodilator/corticosteroid inhalers | Nil | Nil | Nil | Bisphosphonates | Azathioprine | Nil | PRN prednisolone.  Iron supplements | Nil |  |

*Abbreviations: CT, computerised tomography; DLCO,* *diffusing capacity of the lung for carbon monoxide; ND, not done; PRN, pro re nata (as required)*

| **Supplementary Table 4: Genotypes and laboratory findings of affected members of Families A-C** | | | | | | | | | |
| --- | --- | --- | --- | --- | --- | --- | --- | --- | --- |
|  | | | **Family A** | | **Family B** | | **Family C** | | |
|  |  |  | **AII-2** | | **BII-2** | **BI-2** | **CIV-4** | **CII-6** | **CII-8** |
| **Genotype** | | | | | | | | | |
| **Variant** | | | *HCK* p.Y515X | | *HCK* p.Y522F | | *FGR* p.Y523H | | |
| **Zygosity** | | | Heterozygous | | Heterozygous | | Heterozygous | | |
| **Laboratory findings** | | | | | | | | | |
| **Age at time of testing** | | | **Birth**  **(newborn RR)** | **18 months** | **3 years** | **2 years** | **3 years** | **59 years** | **58 years** |
|  | **Units** | **Reference Range** |  |  |  |  |  |  |  |
| **Haematology** | | | | | | | | | |
| Hb | g/L | 115 – 145 | 209 (140-240) | 67 | 117 | 123 | 81 (28 at first presentation) | 143 | 152 |
| RBC | ×10^12^/L | 3.90 – 5.30 | Normal | 2.89 | 4.57 | 5.02 | 3.73 | 4.71 | 4.79 |
| MCV | fL | 75.0 – 87.0 | Normal | 68.0 | 73.1 | 76.4 | 64.9 | 94.5 | 92.6 |
| PLT | ×10^9^/L | 150 – 450 | 113 (150-400) | 235 | 271 | 378 | 364 | 332 | 263 |
| WBC | ×10^9^/L | 4.0 – 11.0 | 29.4 (10-26) | 10.25 | 7.4 | 10.3 | 7.6 | 9.0 | 16.3 |
| Neutrophils | ×10^9^/L | 1.5 – 8.5 | 15.9 (2.7-14.4) | 4.77 | 2.6 | 1.85 | 2.58 | 6.0 | 11.4 |
| Lymphocytes | ×10^9^/L | 2.0 – 9.5 | Normal | 4.53 | 5.01 | 7.93 | 4.3 | 2.1 | 2.9 |
| Monocytes | ×10^9^/L | 0.3 – 1.5 | Normal | 0.82 | 0.53 | 0.41 | 0.59 | 0.7 | 1.3 |
| Eosinophils | ×10^9^/L | 0.1-1.1 | Normal | 0.11 | 0.19 | ND | 0.1 | 0.2 | 0.6 |
| Basophils | ×10^9^/L | 0.00-0.20 | Normal | 0.02 | 0.05 | ND | 0.1 | 0.0 | 0.2 |
| Direct antiglobulin test | NA | Neg | ND | Neg | ND | ND | Neg | ND | ND |
| **Coagulation** | | | | | | | | | |
| PT | seconds | 9.6 – 14.7 | 38.8 (28-38) | 9.9 | Normal | Normal | 11.7 | ND | 10.2 |
| INR | NA | <1.1 | ND | 1.0 | ND | ND | 1.0 | ND | 0.8 |
| APTT | seconds | 26 – 35 | 13.6 (28-38) | 29.3 | Normal | Normal | 31.5 | ND | 32.1 |
| TT | seconds | 14 – 19 | ND | 11.6 | ND | Normal | ND | ND | ND |
| Fibrinogen | g/L | 14 – 40 | 1.6 (1.7-4.2) | 2.8 | ND | Normal | ND | ND | ND |
| D-dimer | ng/ml | <500 | 94520 | ND | ND | ND | ND | ND | ND |
| **Biochemistry** | | | | | | | | | |
| Sodium | mmol/L | 133-146 | 140 (133-156) | 139 | 139 | 134 | 141 | Normal | 139 |
| Potassium | mmol/L | 3.5-5.3 | 5.4 (3.4-6) | 4.9 | 3.9 | 4.7 | 3.8 | Normal | 4.9 |
| Calcium | mmol/L | 2.17 – 2.44 | ND | 2.40 | 2.31 | ND | ND | Normal | ND |
| Urea | mmol/L | 0.8-5.5 | 5.4 (0.8-5.5) | 5.3 | 5.0 | 2.8 | 5.3 | Normal | 6.1 |
| Creatinine | umol/L | 25-73 | 65 (27-81) | 27 | 29 | 40 | 24 | Normal | 68 |
| CRP | mg/L | <20 | <4 (<10) | <5 | <5 | ND | 7.7 | 18 | 33 |
| ESR | mm/hr | 0 – 10 | Normal | 10 | 6 | 30 | 10 | ND | 28 |
| SAA | mg/L | <10 | ND | <3.5 | <3.5 | ND | ND | ND | ND |
| Ferritin | μg/L | 8.6-74.0 | 270 (25-200) | 13 | ND | ND | 120 | 235 | ND |
| Albumin | g/L | 35 - 52 | Normal | 44 | 41 | 36 | 44 | 40 | 40 |
| Total bilirubin | μmol/L | <18 | Normal | 6 | 6 | ND | 3 | 6 | 5 |
| ALT | U/L | 5 – 45 | Normal | 26 | 23 | 16 | 11 | 24 | 1 |
| ALP | U/L | 150 – 380 | Normal | 137 | 191 | ND | ND | 92 | 101 |
| Iron | umol/L | 3-23 | ND | ND | ND | ND | 4 | ND | ND |
| Transferrin | umol/L | 2.2-3.37 | ND | ND | ND | ND | 3.58 | ND | ND |
| Transferrin saturation | % | 20-50 | ND | ND | ND | ND | 4 | ND | ND |
| **Autoantibodies** | | | | | | | | | |
| Rheumatoid factor | IU/ml | 0-15.9 | ND | Neg | ND | ND | ND | Neg | Neg |
| ANA | IU/ml | 0-1.99 | Neg | Neg | ND | Neg | Neg | Neg | Neg |
| ENA | IU/ml | <10 | Neg | Neg | ND | ND | Neg | Neg | Neg |
| ANCA – PR3 | IU/ml | 0-3.49 | Neg | Neg | <0.6 | ND | Neg | Neg | Neg |
| ANCA – MPO | IU/ml | 0-3.49 | Neg | Neg | 0.2 | ND | Neg | Neg | Neg |
| Anti-GBM | IU/ml | <10 | ND | Neg | <1.5 | ND | Neg | ND | ND |
| ACE | IU/L | 0-90 | ND | ND | ND | ND | ND | ND | ND |
| Anti-C1q | IU/ml | <15 | ND | ND | 11 | ND | ND | Neg | ND |
| **Immunology** | | | | | | | | | |
| C3 | g/L | 0.75-1.65 | Normal | 1.41 | 1.25 | Normal | 1.12 | 1.46 | 1.73 |
| C4 | g/L | 0.14-0.54 | Normal | 0.22 | 0.12 | Normal | 0.17 | 0.24 | 0.41 |
| C1Q | g/L | 0.5-2.50 | Normal | ND | ND | Normal | ND | Normal | ND |
| IgG | g/L | 4.9 – 16.1 | Normal | 6.43 | ND | Normal | 5.86 | ND | ND |
| IgA | g/L | 0.4 – 2.0 | Normal | 1.36 | ND | Normal | 0.48 | ND | ND |
| IgM | g/L | 0.5 – 2.0 | Normal | 1.31 | ND | Normal | 0.78 | ND | ND |
| IgD | KU/L | 2 - 100 | ND | 10.0 | ND | ND | ND | ND | ND |
| IgE | KU/L | <52 | ND | 10.3 | ND | Normal | ND | ND | ND |
| RAST/allergen testing | NA | NA | ND | ND | ND | ND | Normal | ND | ND |
| Complement function - classical pathway | % | >= 40% | ND | 101 | 107 | ND | ND | ND | ND |
| Complement function– alternative pathway | % | >=10 | ND | 61 | 64 | ND | ND | ND | ND |
| **Interferon stimulated genes** | | | | | | | | | |
| *CXCL10* | AU | 0.28-5.31 | ND | Normal | 0.26 | ND | ND | ND | ND |
| *CXCL9* | AU | 0.28-2.39 | ND | Normal | 0.24 | ND | ND | ND | ND |
| *IFI27* | AU | 0.09-2.24 | ND | Normal | 12.83 | ND | ND | ND | ND |
| *IFI44L* | AU | 0.24-7.18 | ND | Normal | 1.01 | ND | ND | ND | ND |
| *IFIT1* | AU | 0.17-5.84 | ND | Normal | 0.48 | ND | ND | ND | ND |
| *IFNB1* | AU | 0.23-14.12 | ND | Normal | 0.25 | ND | ND | ND | ND |
| *IFNG* | AU | 0.33-2.59 | ND | Normal | 0.39 | ND | ND | ND | ND |
| *IL-18* | AU | 0.67-1.30 | ND | Normal | 0.34 | ND | ND | ND | ND |
| *RSAD2* | AU | 0.19-6.86 | ND | Normal | 0.39 | ND | ND | ND | ND |
| *SIGLEC1* | AU | 0.17-4.66 | ND | Normal | 0.09 | ND | ND | ND | ND |
| **Microbiology** | | | | | | | | | |
| Blood cultures | NA | NA | Neg | ND | Neg | ND | ND | ND | ND |
| Congenital infection screen | NA | NA | Neg | ND | Neg | ND | ND | ND | ND |
| **Other** | | | | | | | | | |
| Urinalysis (dipstick) | NA | NA | ND | Normal | Normal | ND | Normal | Normal | Normal |
| Urine PCR | mg/mmol creatinine | <15 | ND | 20-93 | ND | ND | 21.6 | ND | ND |

*Abbreviations: ACE, angiotensin-converting enzyme; ACR, albumin-creatinine ratio; ALP, alkaline phosphatase; ALT, alanine aminotransferase; ANA, antinuclear antibody; ANCA – MPO, antineutrophil cytoplasmic antibody – myeloperoxidase; ANCA – PR3, antineutrophil cytoplasmic antibody – proteinase 3; Anti-C1q, anti-C1q antibody; Anti-GBM, anti-glomerular basement membrane antibody; APTT, activated partial thromboplastin time; AU, arbitrary units; C1Q, complement component 1Q; C3, complement component 3; C4, complement component 4; CRP, C-reactive protein; CXCL10, C-X-C motif chemokine ligand 10; CXCL9, C-X-C motif chemokine ligand 9; DLCO, diffusing capacity of the lung for carbon monoxide; ENA, extractable nuclear antigen; ESR, erythrocyte sedimentation rate; FBC, full blood count; Ferritin, ferritin; Hb, haemoglobin; IFI27, interferon-inducible protein 27; IFI44L, interferon-inducible protein 44-like; IFIT1, interferon-induced protein with tetratricopeptide repeats 1; IFNB1, interferon beta 1; IFNG, interferon gamma; Ig, immunoglobulin; IL-18, interleukin 18; INR, international normalized ratio; IU/ml, international units per millilitre; MCV, mean corpuscular volume; MPO, myeloperoxidase; NA, not applicable; ND, not determined; PCR, polymerase chain reaction; PLT, platelets; PR3, proteinase 3; PT, prothrombin time; RA, rheumatoid arthritis; RAAS, renin-angiotensin-aldosterone system; RAST, radioallergosorbent test; RBC, red blood cells; RSAD2, radical S-adenosyl methionine domain containing 2; SAA, serum amyloid A; SIGLEC1, sialic acid-binding Ig-lectin 1; TT, thrombin time; U/L, units per liter; umol/L, micromoles per litre; WBC, white blood cell.*

**Supplementary Figure 1: Co-segregation testing and Sanger chromatograms**


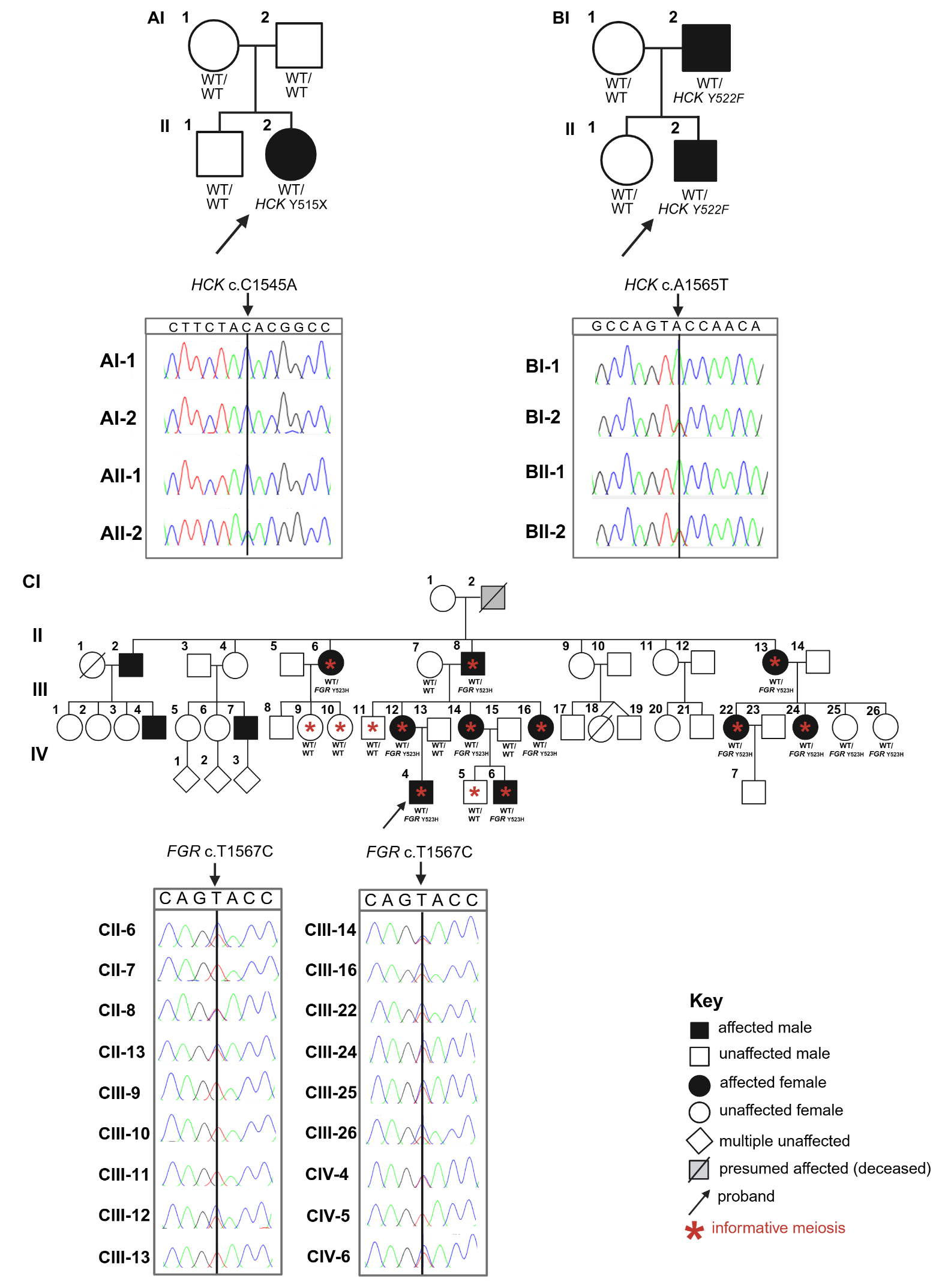

Supplement: Supplementary file 1 — Supplementary Material 1 (DOCX 530 KB) [file 10875_2026_1998_MOESM1_ESM.docx]
